# Developing PMMA/Coffee Husk Green Composites to Meet the Individual Requirements of People with Disabilities: Hip Spacer Case Study

**DOI:** 10.3390/jfb14040200

**Published:** 2023-04-05

**Authors:** Ahmed Fouly, Ibrahim A. Alnaser, Abdulaziz K. Assaifan, Hany S. Abdo

**Affiliations:** 1 Mechanical Engineering Department, College of Engineering, King Saud University, Riyadh 11421, Saudi Arabia; ianaser@ksu.edu.sa; 2 The King Salman Center for Disability Research, Riyadh 11421, Saudi Arabia; aassaifan@ksu.edu.sa; 3 Department of Production Engineering and Mechanical Design, Faculty of Engineering, Minia University, Minya 61519, Egypt; 4 Center of Excellence for Research in Engineering Materials (CEREM), King Saud University, P.O. Box 800, Riyadh 11421, Saudi Arabia; habdo@ksu.edu.sa; 5 King Abdullah Institute for Nanotechnology, King Saud University, P.O. Box 2455, Riyadh 11451, Saudi Arabia; 6 Biomedical Technology Department, College of Applied Medical Sciences, King Saud University, Riyadh 12372, Saudi Arabia; 7 Mechanical Design and Materials Department, Faculty of Energy Engineering, Aswan University, Aswan 81521, Egypt

**Keywords:** PMMA composite, biocomposites, coffee husk filler, artificial hip joint

## Abstract

When replacing a damaged artificial hip joint, treatment involves using antibiotic-laced bone cement as a spacer. One of the most popular materials used for spacers is PMMA; however, it has limitations in terms of mechanical and tribological properties. To overcome such limitations, the current paper proposes utilizing a natural filler, coffee husk, as a reinforcement for PMMA. The coffee husk filler was first prepared using the ball-milling technique. PMMA composites with varying weight fractions of coffee husk (0, 2, 4, 6, and 8 wt.%) were prepared. The hardness was measured to estimate the mechanical properties of the produced composites, and the compression test was utilized to estimate the Young modulus and compressive yield strength. Furthermore, the tribological properties of the composites were evaluated by measuring the friction coefficient and wear by rubbing the composite samples against stainless steel and cow bone counterparts under different normal loads. The wear mechanisms were identified via scanning electron microscopy. Finally, a finite element model for the hip joint was built to investigate the load-carrying capacity of the composites under human loading conditions. The results show that incorporating coffee husk particles can enhance both the mechanical and tribological properties of the PMMA composites. The finite element results are consistent with the experimental findings, indicating the potential of the coffee husk as a promising filler material for enhancing the performance of PMMA-based biomaterials.

## 1. Introduction

Total replacement surgery is a popular treatment for hip joint deterioration for patients with disabilities; however, the artificial joint may require maintenance after a period. The two-stage replacement approach is the preferred treatment for infected total hip replacements. The first stage considers the cleaning and possible removal of the deteriorated hip joint, followed by administering antibiotics at the infection location. The second stage is reimplantation of the hip joint after the infection is eliminated. During the time interval between the stages, antibiotics are administered through antibiotic-infused bone cement, typically in the form of a spacer to maintain proper alignment and prevent soft tissue shortening [1].

To create the spacer, orthopedic departments use a simple molding form to create a reproducible spacer, which can be molded during surgery and tailored to the patient’s specific needs by varying the type and amount of antibiotics used. However, the strength of the bone cement is limited, and spacer failure is a potential complication [2]. There are many studies in the literature regarding the use of spacers [3,4]; however, to date, only a limited number of authors have examined the mechanical strength of PMMA (polymethyl methacrylate) as a spacer material [5]. Polymethyl methacrylate (PMMA) is a widely used material in biomedical applications due to many advantages [6]. PMMA parts are traditionally prepared by mixing PMMA powder with a monomer called methyl methacrylate (MMA) that comes in a liquid phase and then by placing it in preshaped molds [7]. One of the main shortcomings of PMMA is its surface properties, which make it unsuitable for use in friction-based applications. Additionally, PMMA has poor mechanical properties, such as low impact and flexural strengths [8], which can be a drawback in certain situations. When used as a spacer material, PMMA is exposed to various types of stress, such as shear, friction, and compressive stresses. Such stresses can cause spacer fracture and increase the wear rate, which can lead to further suffering for patients with disabilities [9]. One of the most common issues with PMMA parts is the sudden fracture, which causes discomfort to the patient, additional healing costs, and prolonged recovery time [10].

In biomedical materials, the most important demands are typically an appropriate level of strength, stiffness, and wear resistance [11]. To overcome the limitations of PMMA, researchers are trying to find ways for enhancing PMMA properties through different modifications. One such method is chemical modification, which can enhance the impact resistance of PMMA through the use of plasma technology, and the addition of different agents can improve the cross-linking of PMMA [12]. Another approach that researchers have utilized to improve PMMA properties is reinforcing it with different fillers [13]. Following composite strategy, Alhareb et al. [14] investigated the fracture toughness, impact strength, and hardness of PMMA after adding different weight fractions of nitrile butadiene rubber and ceramic materials, such as AL_2_O_3_ and YSZ, in addition to the addition of silane agent. The results showed an improvement in all mechanical characteristics, and the best mixture was found to be 7.5% NBR, 2.5% Al_2_O_3_, and 2.5% YSZ. Nevertheless, they noticed that the PMMA composite had a significant difference in weight compared to pure PMMA, as a result of the high loading of the added materials, particularly ceramic fillers. In an earlier study, Asar et al. [15] explored the impact of incorporating various metal oxides on the mechanical characteristics of heat-cured PMMA resin. They found that adding these metal oxides to PMMA was able to improve the PMMA fracture toughness.

The use of natural materials as fillers in composites has gained attention among researchers due to their abundance in nature [16], low cost, simple manufacturing process, compatibility with living organisms, and environmentally friendly properties [17,18,19]. It has been claimed that incorporating miswak fiber with different lengths, 2, 6, and 12 mm, and with different weight fractions, 3, 6, and 9 wt.%, into the PMMA matrix can enhance the physical and mechanical properties of PMMA composites [20]. Another study investigated reinforcing PMMA with miswak and bamboo fibers, and the researchers recorded enhancement in the compression strength upon increasing additive fiber load fraction. Other researchers tried to add elastomers to the thermoplastic to enhance toughness with the utilization of natural materials [21,22]. Coffee is a widely consumed drink that has become increasingly important in the commercial world over the past century and a half [23]. Coffee production results in a significant amount of agricultural waste, which can make up 30–50% of the total weight of the coffee produced, depending on the processing method [24]. This waste includes coffee pulp and husks, which are among the largest solid byproducts of coffee processing. Until recently, these agricultural wastes had not been put to profitable use, and their disposal was considered harmful to the environment [25]. In recent years, there has been a focus on finding alternative uses for coffee industrial waste to reduce its environmental impact [26], but these efforts have yet to yield practical solutions to effectively utilize this waste.

In the current study, we clarified the need to promote a new material, which could be utilized as a spacer and has satisfactory mechanical characteristics. Thus, the authors chose to use Poly(methyl methacrylate) (PMMA) as it is a popular choice for spacers in various applications. Given the benefits and potential uses of natural materials, further research is needed to determine the mechanical performance of PMMA composites made with natural materials. Therefore, in this study, the authors selected to use coffee husk as the raw material. To the best of their knowledge, there are no existing data on the conversion of coffee husk from a waste product into a usable filler for polymer composites. Additionally, there is no information available on using coffee husk without any chemical treatment, which may incur additional costs and detract from the concept of making use of the waste material. Therefore, the current study includes a complete production process for creating a coffee husk filler without any chemical treatment. The study evaluated the mechanical and tribological properties of PMMA composites with varying weight fractions of coffee husk particles, including hardness, compressive strength, and modulus of elasticity. Additionally, an FEM of the hip implant was created, incorporating the spacer with the mechanical properties obtained from the experiments to estimate the contact stress on the composite samples caused by the human weight. Eventually, the tribological properties of the produced composite were evaluated experimentally under different working conditions, and the worn surfaces were evaluated to identify the wear mechanism.

## 2. Materials and Methods

### 2.1. Materials 

In the current study, a self-curing acrylic resin purchased from Protechno Famadent S.L.U. in Spain is used for quickly repairing and relining denture plates made from acrylic material. It is also suitable for hydroflasking both partial and full dentures. The resin comes in two parts: a white powder made of Poly(methyl methacrylate) or PMMA with a density of 1.18 g/cm^3^ and a colorless liquid monomer composed of methyl methacrylate (MMA) with a density of 0.94 g/cm^3^. According to the manufacturer’s data sheet, it has a flexure strength of 65.8 MPa and a Young’s modulus of 2263 MPa. The coffee husks were first ground into smaller pieces using a mortar and pestle. Then, a grain miller was used to pulverize the pieces at 200 RPM. The obtained powder was switched to the ball-milling jar (Pulverisette 7, Fritsch, Idar-Oberstein, Germany). The machine was set to rotate at 200 RPM, and the milling process lasted 8 h, with 15 milling and 15 min rests to prevent excessive heat buildup in the jar.

### 2.2. Fabrication of PMMA Composites

**Figure 1:**
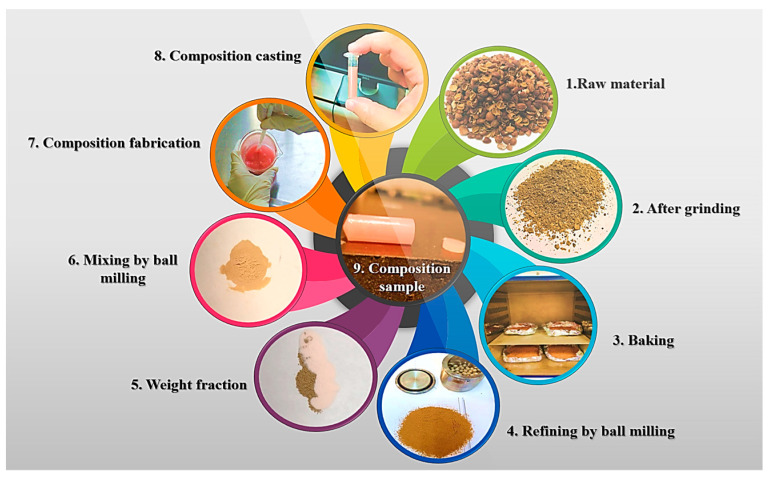
Flowchart of PMMA–CH composite sample preparation.

To prepare the samples, the appropriate amounts of dry PMMA and coffee husk powders were weighed according to the desired weight ratios of 0, 2, 4, 6, and 8 wt.%. The powders were then mixed in a ball-milling machine for 5 min to ensure a homogenous dispersion of the coffee husk particles within the PMMA powder. Afterward, the liquid monomer was added in a weight ratio of 5 to 3.5, solid to liquid, for all samples. The weight of the solid powder was calculated by summing the weight of the PMMA and coffee husk powders. The mixture was then mixed manually for 25 s at a temperature of 28 °C and humidity of 55%. When the mixture became sticky, like dough, after around 20 to 30 s of mixing, it was placed in a cylindrical die of 25 mm × 8 mm. After 30 min, the samples were fully hardened and were removed from the molds. These procedures were carried out according to the PMMA manufacturer’s data sheet recommendations and instructions. As previously mentioned, the PMMA/CH composites were produced with coffee husk weight fractions of 0, 2, 4, 6, and 8 wt.% and are referred to in this work as PMCH0, PMCH2, PMCH4, PMCH6, and PMCH8, respectively. Figure 1 illustrate the production process of the PMMA–CH composite samples.

### 2.3. Characterization and Testing

The X-ray diffraction pattern (XRD) analysis utilizing D8 discover equipment from Bruker in Germany was employed to assess the crystallinity of the coffee husk powder. In addition, field emission scanning electron microscopy was used to observe the morphology of the produced powder, JEOL JSM-7600F, Tokyo, Japan. The PMMA/coffee husk composite’s crystallinity was also assessed using XRD. To determine the thermal decomposition of the PMMA composites, a thermogravimetric TGA was conducted on the TA-Q500 System of TA, using about 10 mg of each composite sample. The TG-DTA was used to heat the samples from 30 to 800 °C, at a heating rate of 10 °C/min, under a nitrogen atmosphere. The equipment used for this analysis was the STA 449 F3 model from NETZSCH Germany.

The strength and durability of the PMMA/CH composite were evaluated using a combination of hardness and compression tests. The surface hardness of the composite specimens was evaluated using Shore hardness D, which applies 5 ± 0.5 kg load for a dwell time of 15 s based on ASTM D2240 [27]. The measurements were taken on the surface of the samples six times, and the average value was calculated, considering the standard errors. The samples were also tested on a computer-controlled servohydraulic universal testing machine, which has a capacity of 30 tons and a strain rate of 1 min^−1^. The compression experiments were undertaken based on ASTM: E9-89a [28] using Instron 5582 Microtester (Instron, University Ave, Norwood, USA). The results from the tests were used to determine the composite’s mechanical properties, including Young’s modulus and the compressive yield strength.

**Figure 2:**
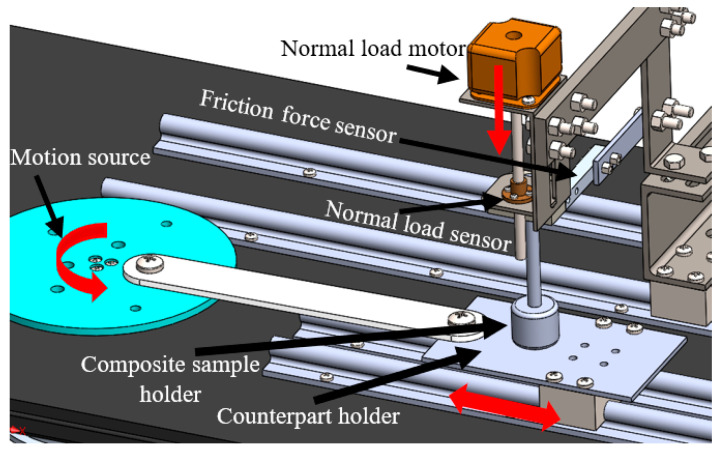
Reciprocating pin-on-disk tribometer [7].

The tribological tests were conducted to investigate the tribological characteristics of PMMA/coffee husk composites under dry sliding conditions at 27 °C and 60% relative humidity. A reciprocating pin-on-disk tribometer, shown in Figure 2, was utilized where an 8 mm diameter and 25 mm length PMMA/coffee husk pin slides against a rectangular disk made of stainless steel or a part of cow bone, and the tests were conducted according to ASTM G99-95 [29]. The study seeks to evaluate the tribological properties of the proposed PMMA composite as a spacer, and, consequently, a real cow bone was used during the tribology test. Before each experiment, the stainless steel and cow bone surfaces were cleaned using acetone and dried with a heat gun to eliminate any contaminants. Additionally, the PMMA/coffee husk composite specimens were ultrasonically washed with acetone and dried before starting every experiment. The friction experiments were conducted under fixed sliding speed of 0.4 m/sec, using different normal loads of 5, 10, 15, and 20 N. The wear of each specimen was determined by calculating the weight difference before and after the test. To ensure accurate results, each experiment was repeated five times under the same load conditions, and the average was determined while considering the standard errors. Following the tribological experiments, the morphology of the rubbed surfaces was evaluated with the aid of an SEM microscope (JCM-6000Plus; JEOL, Tokyo, Japan). To improve the samples’ conductivity, a thin layer of platinum was applied to all surfaces of the composite samples before conducting the scanning.

### 2.4. Finite Element Model

**Figure 3:**
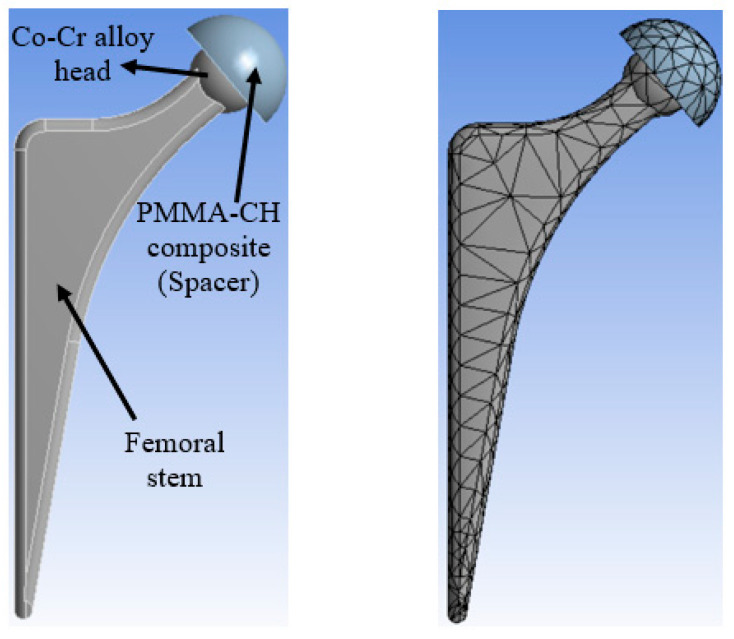
A schematic of the PMMA–CH composite spacer on a hip joint and the finite element model with meshing. Adapted from [18].

To examine the ability of the new PMMA–CH composite to support weight when used as a spacer, a finite element model was created. In accordance with study by Ishihara et la., they assumed that the hip joint could be divided into two main parts: the femoral head covered by an acetabular liner, Figure 3, which in our case will represent the hip spacer. In the current study, ANSYS software was used to model the spacer of a hip joint. In the finite element model, a hip joint ball was bound to the hip joint stem. The properties of the spacer were inserted to the software based on the new PMMA–CH composite’s characteristics.

The boundary conditions of the proposed model in addition to the loading conditions were applied based on a previous study conducted by Bergmann et al. [30]. In this study, the authors showed that the surface of a hip joint subjected to a combination of forces, and these forces can be defined with 3 components in the 3D directions based on the motion type. Assuming a human body has 100 kg, the maximum forces for standing position are 1540, 3480, and 70 N in the x, y, and z direction, respectively. Meanwhile, the ball head and the stem were totally fixed in the x, y, and z directions. The interaction between the ball and the spacer was set as bonded. The measured properties of the PMMA–CH composites were inserted into the software based on experimental testing outcomes. The ball of the femoral head was assumed to made of Co–Cr alloy. The model was meshed automatically; consequently, it consisted of elements in the shape of hexahedron and tetrahedron elements. Based on the automatic mech, the model was divided into 1247 elements with 2525 nodes.

## 3. Results and Discussion

**Figure 4:**
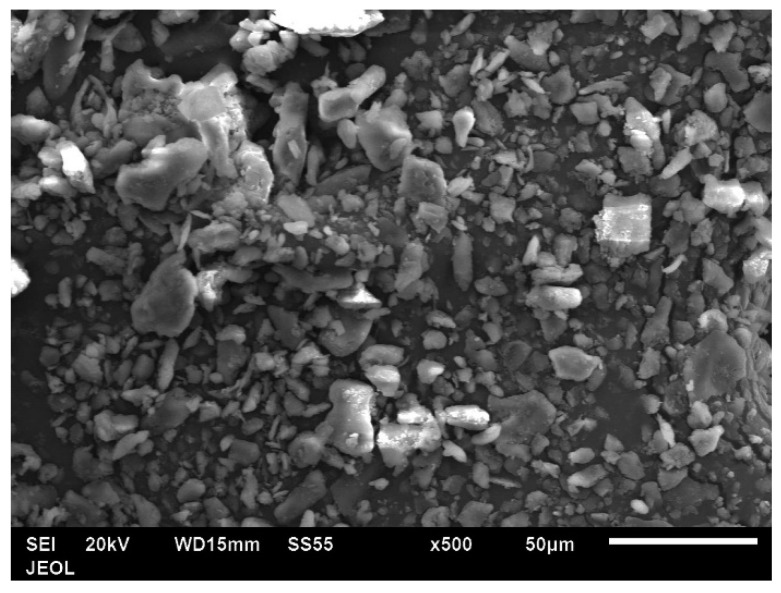
SEM image of coffee husk particles.

As previously indicated, the coffee husk powder was ground in two stages. The final condition of the coffee husk powder is shown in Figure 4, which displays an SEM image of the coffee husk particle morphology. The date pit particles have an irregular, equiaxed shape and a rough surface. Additionally, some particles were found to have significantly smaller sizes and were observed to be clustered.

**Figure 5:**
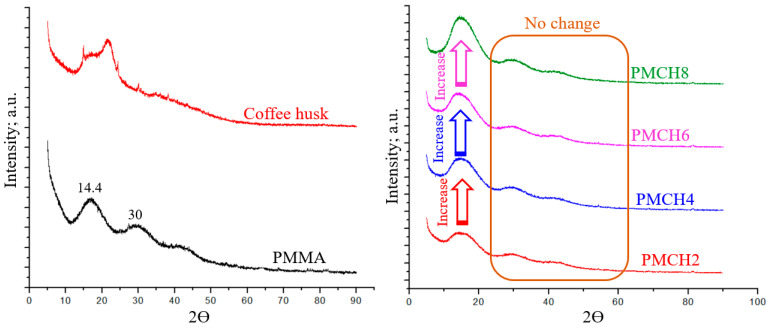
XRD of pure PMMA, coffee husk, and PMMA/coffee husk composites.

To analyze the chemical composition of the PMMA/coffee husk composites, X-Ray Diffraction (XRD) was employed. Figure 5 depicts the XRD analysis of the pure PMMA powder, the prepared coffee husk powder, and the PMMA/coffee husk composites. 

The XRD analysis of pure PMMA showed three broad peaks with a high intensity at 2θ of 14.4 and a low intensity at 30 and 41.8, which were also observed for all PMMA/coffee husk composites. These peaks indicate the amorphous nature of the PMMA polymer [31]. These observations are consistent with the results presented by Hashem et al. [32] for PMMA. The XRD pattern of the prepared coffee husk showed two main well-defined peaks. The first peak with a low intensity at 2θ of 15, which corresponds to the crystallographic plane (110) [33], and the second peak with a high intensity at 2θ of 22.02 corresponds to the crystallographic plane (200) [34]. The XRD analysis of the coffee husk shows an ordered structure that is identical with cellulose type I, which has Ic of 33.04% [35]. The XRD analysis of the PMMA/coffee husk composites showed that the addition of the coffee husk did not affect the structural properties of PMMA, suggesting that there was no chemical reaction between the two components and that the coffee husk was only physically mixed with PMMA.

**Figure 6:**
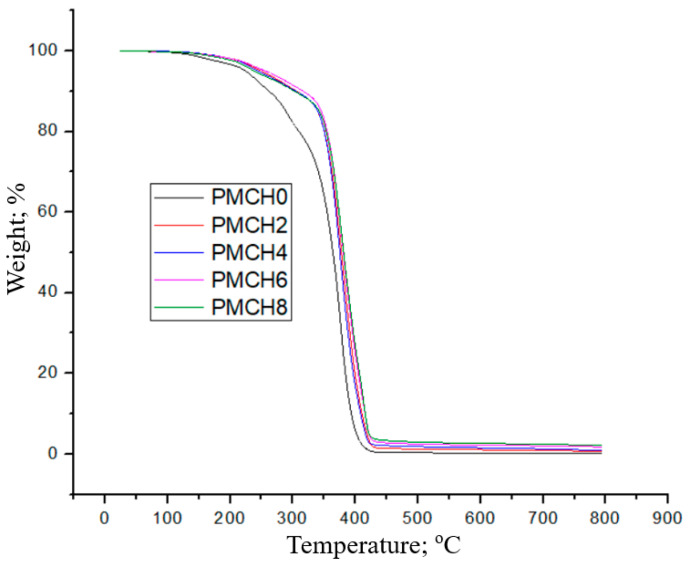
TGA of pure PMMA and PMMA/coffee husk composites.

Figure 6 demonstrates the thermal stability of both PMMA and PMMA/coffee husk composites by applying TGA analysis. The results indicate that PMMA and its composites started to lose weight at 150 °C, and about 90% of the weight loss occurs at 420 °C, which shows that the incorporation of coffee husk into the PMMA has a negligible effect on PMMA’s thermal stability. These results could be attributed to the addition of a low-weight fraction of coffee husk, which would not significantly impact the thermal characteristics of the composite. However, a slight enhancement in thermal stability is observed by adding 8 wt.% coffee husk. This increase in stability could be due to the increased number of active sites, which could raise the decomposition temperature of the composite [36].

**Figure 7:**
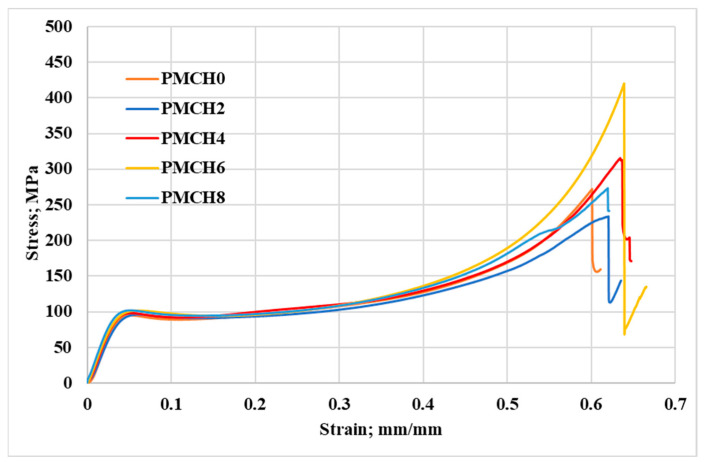
Compressive stress–strain diagram of pure PMMA and PMMA/coffee husk composites.

**Figure 8:**
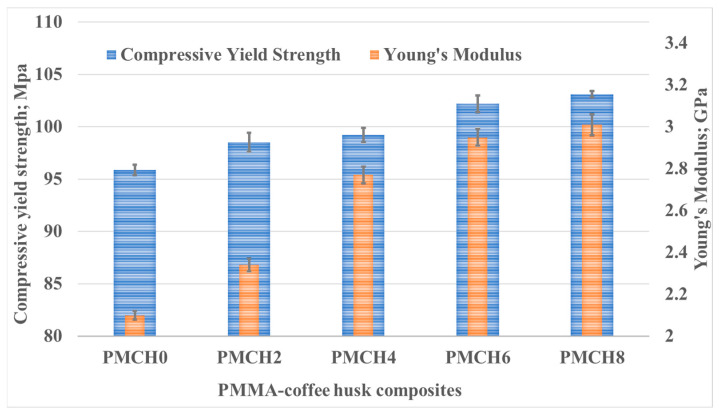
Compressive yield strength and Young’s modulus of PMMA/CH composites.

**Figure 9:**
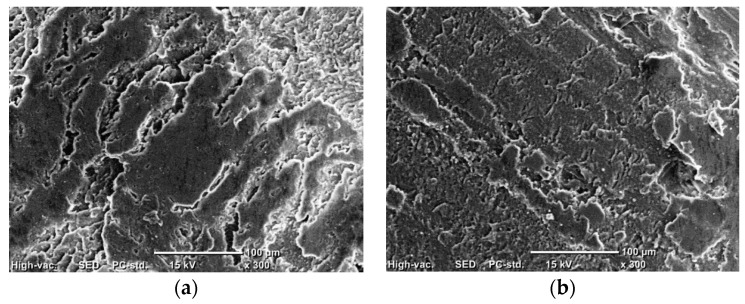
SEM of the fracture surface due to compression test, (**a**) PMCH0 and (**b**) PMCH8.

During the compression tests of PMMA composites with varying weight fractions of coffee husk, the stress and corresponding strain were recorded, as shown in Figure 7. The results reveal that the incorporation of coffee husk in different loading fractions had a significant impact on the compressive characteristics of PMMA, which led to an enhanced performance compared to pure PMMA. Figure 8 illustrates the effect of the coffee husk incorporation on the Young’s modulus and compressive yield strength of the PMMA composites. Increasing the content of coffee husk led to an improvement in the Young’s modulus and compressive yield strength of the PMMA composites. The PMMA composite containing 8 wt.% coffee husk was the best in terms of yield strength. Young’s modulus and compressive yield strength increased by 43.33% and 7.56%, respectively, compared with pure PMMA. The improvement in compressive yield strength can be attributed to the homogenous distribution of coffee husk particles within the PMMA matrix, which enabled the matrix to transfer the compressive load to the coffee husk powder and dissipate it. Additionally, during compression testing of polymers, filler particles can help prevent the propagation of cracks in the matrix as they begin to form [37]. A comparison of the fracture surface of the PMMA composite with 8% coffee husk loading (PMCH8) to pure PMMA, as shown in Figure 9, revealed distinct differences in the fractographic patterns. The fracture surface of pure PMMA showed significant cracks, which can lead to deterioration in the material and a decrease in the mechanical properties. However, the PMCH8 composite showed fewer cracks and a rough surface. The increase in surface roughness indicates that the crack path has been distorted by the presence of the coffee husk particles, which depicts an improvement in the strength of the PMMA/coffee husk composite.

**Figure 10:**
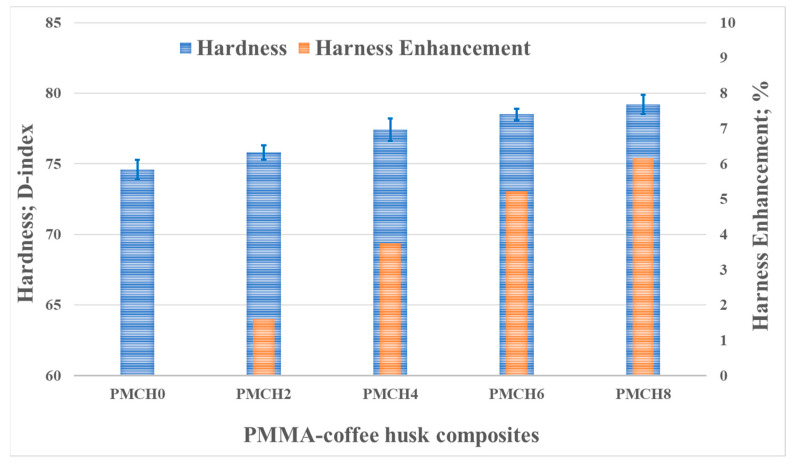
Hardness of PMMA/CH composites.

The hardness of the PMMA composite samples with varying amounts of coffee husk was evaluated using the shore D index, as shown in Figure 10. As the percentage of coffee husk increased, the hardness of the composites also increased. The highest enhancement in hardness, about 6%, was observed for the composite with 8 wt.% coffee husk (PMCH8) compared to pure PMMA. This increase in hardness is attributed to the better distribution of coffee husk particles within the PMMA matrix [38], which improves the intermolecular bonds and load transfer between the matrix and the filler. Additionally, the higher proportion of coffee husk in the composite also results in increased stiffness. Furthermore, the enhancement in hardness can also be attributed to the increase in stiffness due to the increasing proportion of coffee husk.

**Figure 11:**
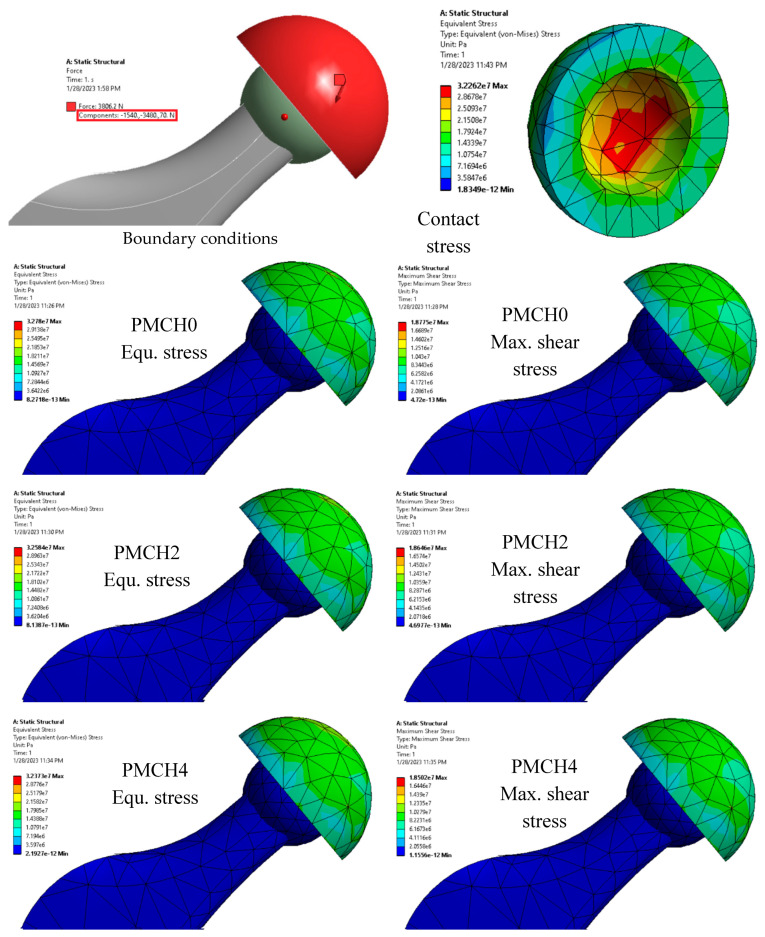

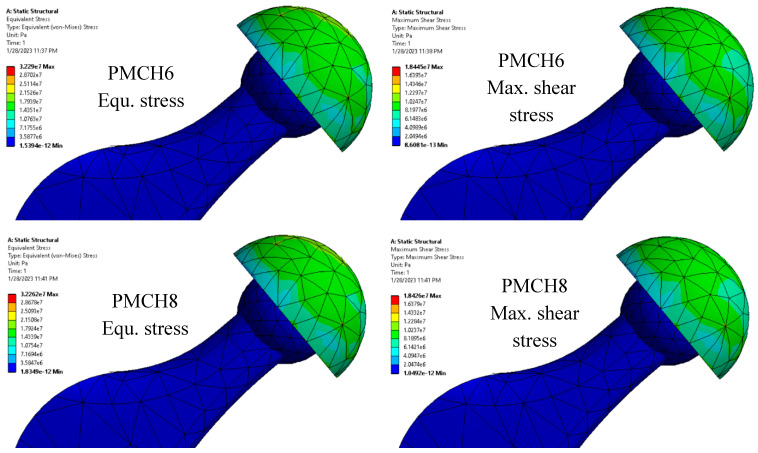
Finite element model with boundary conditions and contact stresses for different PMMA–coffee husk composites. Adapted from [18].

**Figure 12:**
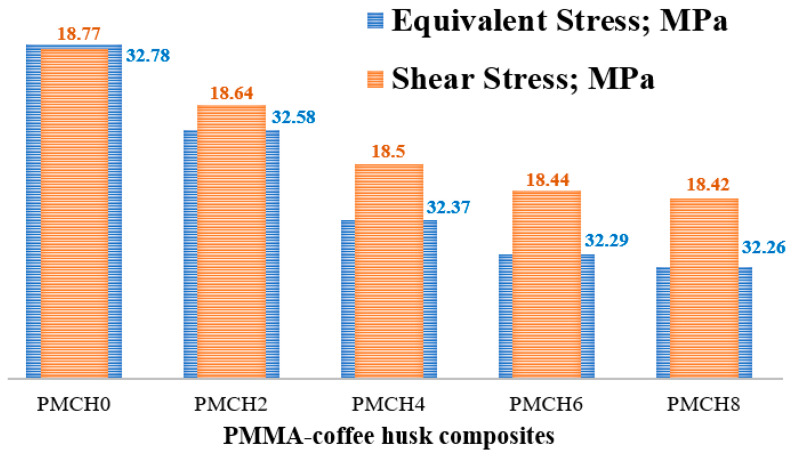
Contact stresses on the surface of the spacer.

It is evident that using coffee husk particles in the PMMA matrix enhances its mechanical properties. As the composite is intended to be used as a spacer, it is crucial to evaluate its load-bearing capability under realistic conditions. Researchers have determined this by assessing the contact stresses on the surface of the parts that come into contact under specific conditions [39]. An ANSYS model of the hip joint incorporating the spacer was created to achieve this. The stresses generated on the spacer surface due a person standing are demonstrated in Figure 11. The specific conditions for these calculations are outlined in Section 2.4 and are shown in the red rectangle in Figure 11. The amount of stress created on the spacer’s outer and inner surfaces is also shown. The greatest amount of stress was observed on the spacer’s inner surface where it is in direct touch with the hip joint ball head. The FE analysis shows that an inconsiderable change in the equivalent stress and the maximum shear stress occurred with the variation of coffee husk weight fraction in PMMA, which affected the mechanical properties, as shown in Figure 12. The increase in the content of coffee husk resulted in a decrease in both generated stresses, which is consistent with the improvement in the measured mechanical characteristics. The decrease in the contact stresses indicates an increase in load-carrying capacity [40]. Additionally, the highest equivalent stress generated is 32.78 MPa, which is roughly half of the ultimate compressive stress as determined in experiments, making it safe to use these new composites to produce the spacer.

**Figure 13:**
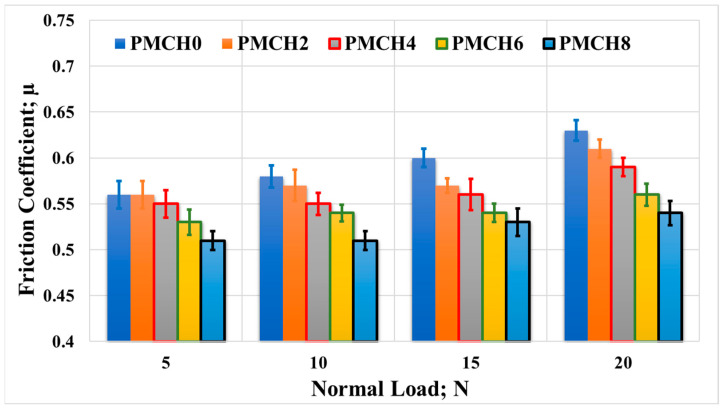
Friction coefficient of the PMMA/coffee husk composites, with different coffee husk concentrations, rubbed against a stainless steel disk under different applied normal loads.

The results from the friction tests are presented in Figure 13, which displays the average coefficient of friction plotted against the normal load applied when rubbing the PMMA/coffee husk composites against a stainless steel disk. The results show that the incorporation of coffee husk led to a reduction in the coefficient of friction compared to pure PMMA. At a normal load of 5 N, PMCH8 displayed the lowest coefficient of friction (0.51), representing a 9% reduction compared to pure PMMA (0.56). With the increase in the applied normal load, the reduction in the coefficient of friction between PMHA0 and PMHA8 ranged from 12% to 14%. Additionally, the friction coefficient gradually increased upon increasing the applied normal load, which could be attributed to the temperature rising at the contact surfaces during the rubbing process [41]. This increase in temperature may increase the adhesion among the contact surfaces [42].

**Figure 14:**
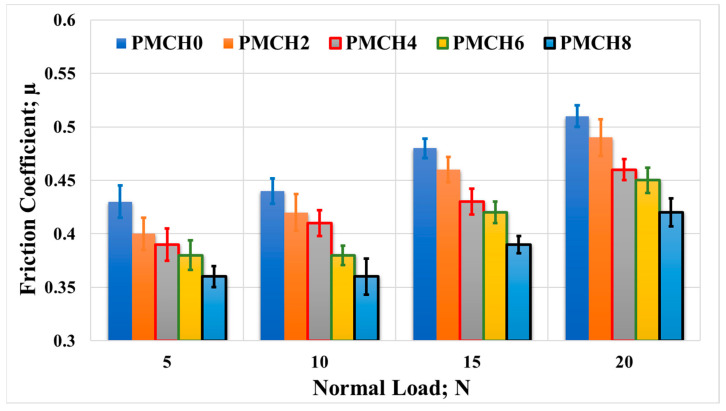
Friction coefficient of the PMMA/coffee husk composites, with different coffee husk concentrations, rubbed against a cow bone disk under different applied normal loads.

The tribological performance of the PMMA/coffee husk composite samples against a cow bone surface was evaluated, and the results are presented in Figure 14. Similar to the performance against the stainless steel disk, the addition of coffee husk reduced the friction coefficient compared to pure PMMA. The lowest coefficient of friction (0.36) was observed for PMCH8 under a normal load of 5 N, indicating a 16.2% decrease compared to pure PMMA (0.43). The difference between PMHA0 and PMHA8 was found to be the maximum (18.7%) when a normal load of 15 N was applied. The friction coefficient also increased with the increase in the applied load.

**Figure 15:**
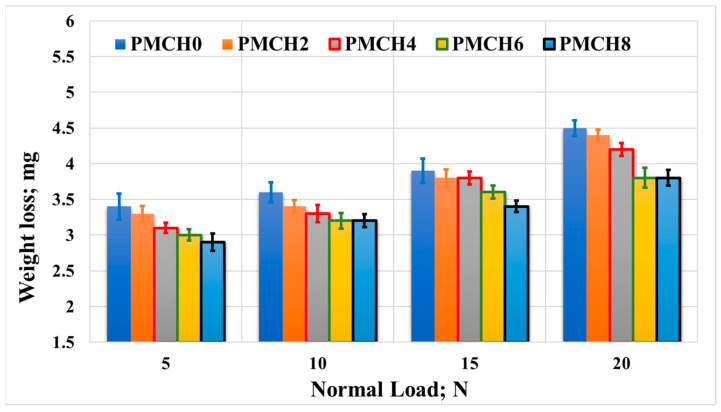
Wear of the PMMA/coffee husk composites, with different coffee husk concentrations, rubbed against a stainless-steel disk under different applied normal loads.

**Figure 16:**
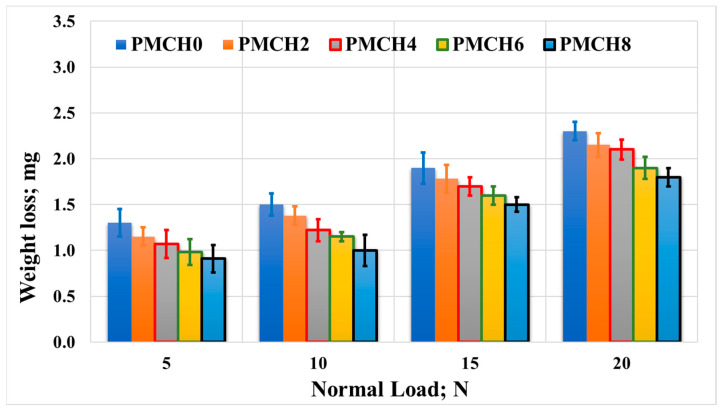
Wear of the PMMA/coffee husk composites, with different coffee husk concentrations, rubbed against a cow bone disk under different applied normal loads.

The impact of incorporating coffee husk on the wear of the PMMA composites was recorded and is presented in Figure 15 and Figure 16. The results reveal that the weight loss of the PMMA composites decreased as the weight fraction of coffee husk increased, which reached 33%. This implies that adding coffee husk filler improved the wear resistance of the PMMA composites. The improvement in mechanical properties discussed earlier could be one of the factors contributing to the enhancement of wear resistance. This could be due to the increase in bonding strength between the coffee husk and PMMA matrix, which enhanced the load-carrying capacity, thereby preventing the PMMA composite sample surface from degrading during the tribological test [43]. Additionally, as the coffee husk loading fraction increased and subsequently increased the hardness of the PMMA composite, the wear resistance may be increased [44]. This is likely due to the improved load-carrying capacity resulting from the stronger bonding between the coffee husk and PMMA matrix, as discussed earlier. These results are in line with the simulation results, which illustrate a decrease in the contact stresses on the spacer surface, which in turn decreased the wear during the frictional process. However, it should be noted that increasing the applied load during the tribological tests led to an increase in weight loss. This is probably due to the high temperature generated during the friction process, which increases the contact area and the frictional force, leading to the breakdown of the PMMA composite surface.

**Figure 17:**
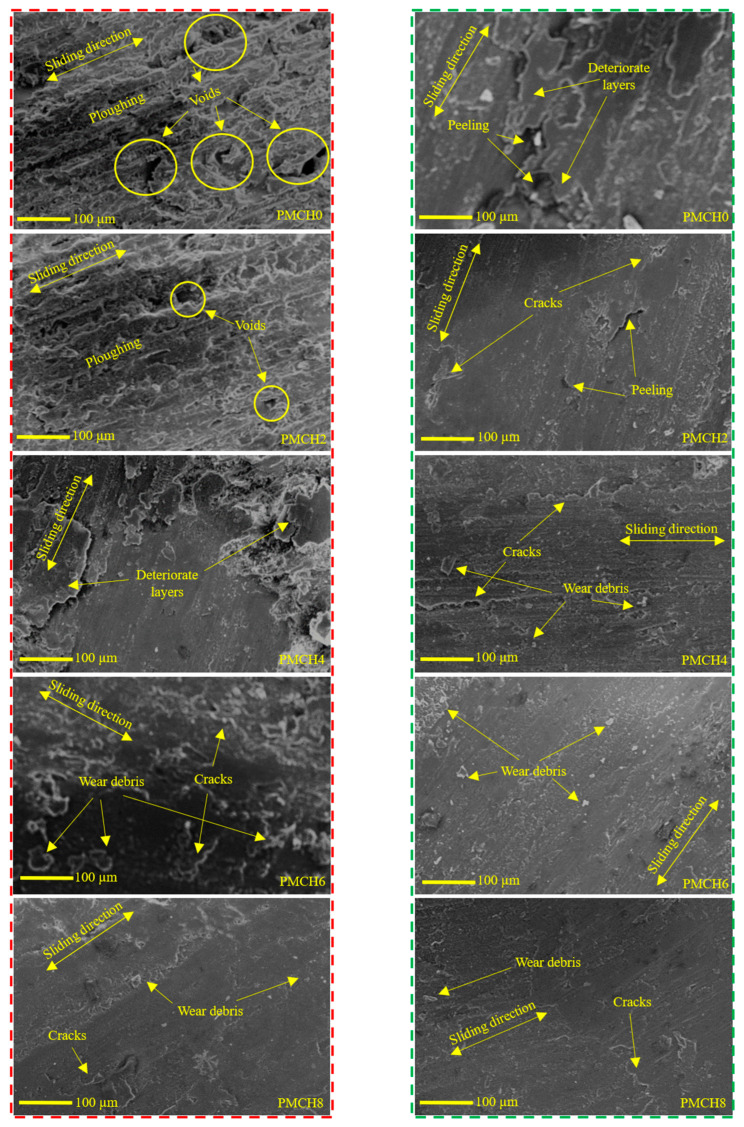
SEM of PMMA composite surfaces, where, against tainless steel are in the red rectangular and against cow bone counterpart are in the green rectangular.

To further understand the improved wear characteristics of the PMMA/coffee husk composites, the worn surfaces of the PMMA composite samples were examined using SEM, as shown in Figure 17. The red rectangle in Figure 17 represents the worn surface of the PMMA/coffee husk composites tested against the stainless-steel disk, while the green rectangle represents the worn surface of the composites tested against the cow bone disk. The worn surface of pure PMMA (PMCH0) showed significant degradation with numerous eliminated and deteriorated layers, large voids, and peeling. This degradation was caused by the plowing of the PMMA surface during the rubbing, leading to a marked increase in weight loss (wear). The eliminated layers were weak, increasing the shear resistance and subsequently the friction coefficient [45]. The wear mechanism is evident as a delamination mechanism, which is known to increase the wear and friction coefficient [46]. The SEM photographs of the PMMA composites containing coffee husk filler show smoother surfaces than pure PMMA, indicating that the coffee husk filler improved the strength of the composite surfaces, reducing wear loss and the friction coefficient of the PMMA composite. However, as the weight fraction of coffee husk increased, changes occurred in the wear mechanism.

When the coffee husk weight fraction reached 4 wt.%, PMCH4 showed damage initiation, microcrack propagation, and pores, and the wear mechanism changed from delamination to fatigue delamination. Increasing coffee husk weight fraction to 6 wt.%, PMCH6 showed a smoother surface with some micro plowing and microcracks, indicating a change in the wear mechanism. Increasing the coffee husk filler to 8 wt.%, PMCH8 exhibited the highest hardness and strength, resulting in inconsiderable cracks and debris on the composite surface, ultimately reducing the wear loss and friction coefficient of the PMMA composite samples.

## 4. Conclusions

The current investigation aims to improve the mechanical and tribological properties of PMMA for use as a spacer in artificial hip joint replacement surgeries. The outcomes of the current study can be summarized as follows:

- Natural powder and composite materials with varying weight fractions of coffee husk were developed.
- The mechanical and tribological properties of the PMMA–coffee husk composites were evaluated.
- An ANSYS model was created to assess the performance of the spacer using the properties determined for the composites.
- The results show a noticeable improvement in the overall mechanical and tribological properties of the PMMA–coffee husk composites.
- The homogenous distribution of coffee husk particles increased the PMMA composite hardness by over 6%.
- Increasing the weight fraction of coffee husk filler up to 8 wt.% gradually improved the compressive yield strength and Young’s modulus by 7.6% and 43%, respectively, compared to pure PMMA.
- The tribological results show that incorporating coffee husk into PMMA decreased the friction coefficient and wear by 18.7% and 33%, respectively.
- The FEA analysis indicated that increasing the coffee husk content in the composite reduced the contact stresses on the spacer, enhancing its load-carrying capacity.

## Data Availability

All data used in this study are declared in the paper.
